# Crosstalk between nonclassical monocytes and alveolar macrophages mediates transplant ischemia-reperfusion injury through classical monocyte recruitment

**DOI:** 10.1172/jci.insight.147282

**Published:** 2021-03-22

**Authors:** Chitaru Kurihara, Emilia Lecuona, Qiang Wu, Wenbin Yang, Félix L. Núñez-Santana, Mahzad Akbarpour, Xianpeng Liu, Ziyou Ren, Wenjun Li, Melissa Querrey, Sowmya Ravi, Megan L. Anderson, Emily Cerier, Haiying Sun, Megan E. Kelly, Hiam Abdala-Valencia, Ali Shilatifard, Thalachallour Mohanakumar, G.R. Scott Budinger, Daniel Kreisel, Ankit Bharat

**Affiliations:** 1 Division of Thoracic Surgery and; 2 Division of Pulmonary and Critical Care Medicine, Feinberg School of Medicine, Northwestern University, Chicago, Illinois, USA.; 3 Department of Surgery, Washington University in St. Louis, St. Louis, Missouri, USA.; 4 Department of Biochemistry and Molecular Genetics, Feinberg School of Medicine, Northwestern University, Chicago, Illinois, USA.; 5 Norton Thoracic Institute, St. Joseph’s Hospital and Medical Center, Phoenix, Arizona, USA.; 6 Department of Pathology and Immunology, Washington University in St. Louis, St. Louis, Missouri, USA.

**Keywords:** Transplantation, Organ transplantation

## Abstract

Primary graft dysfunction (PGD) is the predominant cause of early graft loss following lung transplantation. We recently demonstrated that donor pulmonary intravascular nonclassical monocytes (NCM) initiate neutrophil recruitment. Simultaneously, host-origin classical monocytes (CM) permeabilize the vascular endothelium to allow neutrophil extravasation necessary for PGD. Here, we show that a CCL2-CCR2 axis is necessary for CM recruitment. Surprisingly, although intravital imaging and multichannel flow cytometry revealed that depletion of donor NCM abrogated CM recruitment, single cell RNA sequencing identified donor alveolar macrophages (AM) as predominant CCL2 secretors. Unbiased transcriptomic analysis of murine tissues combined with murine KOs and chimeras indicated that IL-1β production by donor NCM was responsible for the early activation of AM and CCL2 release. IL-1β production by NCM was NLRP3 inflammasome dependent and inhibited by treatment with a clinically approved sulphonylurea. Production of CCL2 in the donor AM occurred through IL-1R–dependent activation of the PKC and NF-κB pathway. Accordingly, we show that IL-1β–dependent paracrine interaction between donor NCM and AM leads to recruitment of recipient CM necessary for PGD. Since depletion of donor NCM, IL-1β, or IL-1R antagonism and inflammasome inhibition abrogated recruitment of CM and PGD and are feasible using FDA-approved compounds, our findings may have potential for clinical translation.

## Graphical abstract

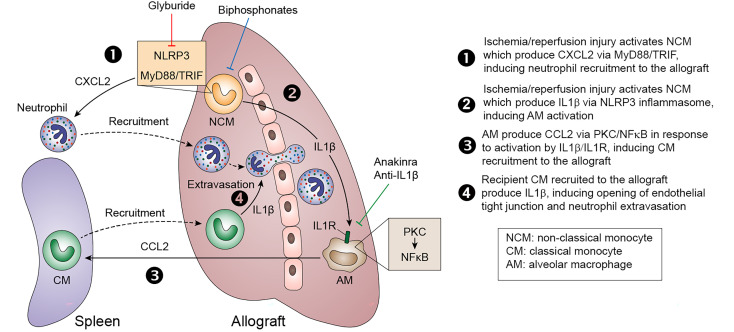

## Introduction

Primary graft dysfunction (PGD), the clinical manifestation of lung transplant ischemia-reperfusion injury, affects over 50% of lung recipients within the first 72 hours and is the predominant cause of short-term mortality, as well as chronic lung allograft rejection (1, 2). Neutrophils are not only the histological hallmarks of transplant ischemia-reperfusion injury, but they also drive its pathogenesis (3, 4). While neutrophil depletion can ameliorate PGD, it is not clinically feasible, given their importance in pathogen clearance. We and others have recently demonstrated that donor human (CD14^lo^CD16^hi^) and murine (CX3CR1^+^Ly6C^lo^CCR2^–^) nonclassical monocytes (NCM) retained in the vascular space of the donor lungs recruit neutrophils to transplanted lungs through the release of CXCL2 (5–8). Genetic and pharmacological depletion of NCM attenuates neutrophil recruitment and prevents PGD. Host-origin Ly6C^hi^CCR2^+^ classical monocytes (CM) are recruited from the spleen to the transplanted lungs, and complementary to the NCM, they mediate extravasation of the recruited neutrophils into the interstitial space by permeabilizing the endothelium through the secretion of IL-1β (9). Therefore, both donor NCM and recipient CM are necessary for neutrophil trafficking into reperfused lung grafts (2).

CM originate in the BM but reside in the spleen, which serves as a monocyte reservoir for rapid deployment of the CM to the site of injury (10). Indeed, the chemokine receptor CCR2, a ligand for CCL2 (also known as monocyte chemoattractant protein 1; MCP-1), has been shown to promote recruitment of CM to the site of injury (11–13). However, mechanisms that drive the recruitment of CM into lung grafts remain unknown. The characterization of mechanisms of monocyte trafficking into the transplanted lung has the potential to not only ameliorate transplant ischemia-reperfusion injury by reducing neutrophil extravasation, but also to reduce long-term allograft rejection and fibrosis by inhibiting development of monocyte-derived profibrotic macrophages (14). Alveolar macrophages (AM) are long-lived cells found in the alveoli and airways, and they have a range of functions in the lungs, including maintenance of homoeostasis and immune surveillance (15). AM induced early reperfusion injury, and neutrophil recruitment has been postulated and verified by several groups (5, 16).

Using the vascularized murine lung transplant model, as well as tissues from human transplant recipients, we found in the present report that donor AM were the predominant secretors of CCL2, which was necessary for recruitment of CM. Intriguingly, 2-photon imaging and multichannel flow cytometry demonstrated that depletion of donor NCM attenuated recruitment of CM to the transplanted lungs. Unbiased transcriptomic profiling revealed that donor NCM produced IL-1β, while AM produced CCL2. Donor NCM, through the release of IL-1β, activated donor AM, which was responsible for the secretion of CCL2, recruitment of CM, and development of PGD. Pharmacological and genetic inhibition of the NLRP3 inflammasome prevented IL-1β release and the activation of donor AM, which prevented CM recruitment and development of PGD. Thus, we demonstrated that crosstalk between donor NCM and donor AM drives the recruitment of recipient splenic CM. Our findings have potential implications in the design of clinically relevant interventions to attenuate PGD and acute and delayed lung allograft dysfunction.

## Results

### Depletion of donor NCM suppresses recruitment of recipient splenic CM.

**Figure 1:**
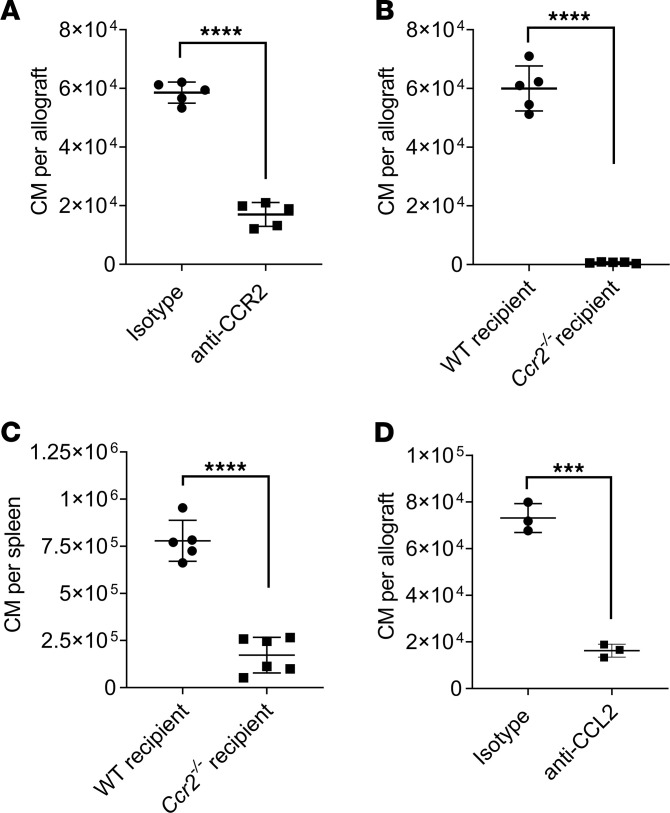
The CCL2-CCR2 axis is necessary for recipient classical monocytes (CM) recruitment to the allograft. (**A**) Flow cytometry quantification of CM (live CD45^+^Ly6G^–^NK1.1^–^CD11b^+^SiglecF^–^CD24^–^Ly6C^hi^) recruited into the allograft after treatment of recipients with i.v. IgG isotype or anti-CCR2 antibodies (*n* = 5). (**B**) Flow cytometry quantification of CM as described in **A**, recruited into the allograft using WT or *Ccr2^–/–^* recipient mice (*n* = 5). (**C**) Flow cytometry quantification of CM (live CD45^+^Ly6G^–^NK1.1^–^CD19^–^CD11b^+^CF4/80^–^CD11c^–^Ly6C^hi^) in spleens of WT or *Ccr2^–/–^* recipients after lung transplant (*n* = 5–6). (**D**) Flow cytometry quantification of CM as described in **A**, recruited into the allograft after treatment of recipients with i.v. IgG isotype or anti-CCL2 antibodies in recipient mice (*n* = 3). Graphs show means ± SD. Graphs were analyzed by unpaired Student’s *t* test. ***P < 0.001; ****P < 0.0001.

**Figure 2:**
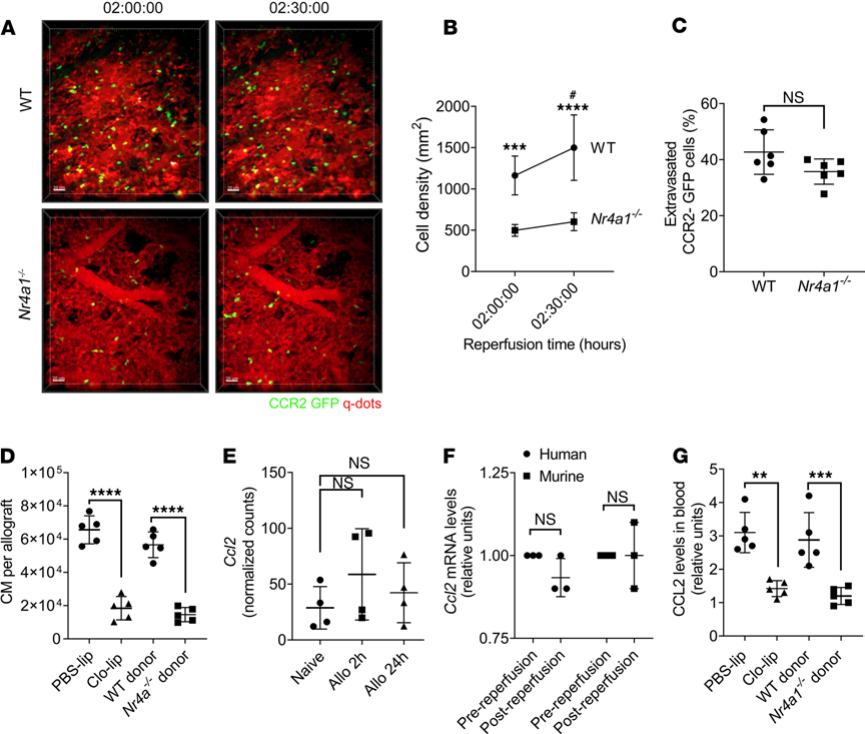
Depletion of donor nonclassical monocytes (NCM) suppresses the recruitment of recipient splenic classical monocytes (CM) to the allograft. (**A**–**C**) Intravital 2-photon imaging between 2 and 2.5 hours after reperfusion. (**A**) Representative still images of WT and *Nr4a1^–/–^* donor grafts. Green, CCR2-GFP; red, Qdot655 blood vessels. (**B** and **C**) CCR2-GFP cell density (**B**) and percent of extravasated CCR2-GFP (**C**) calculated using NIH ImageJ software in WT and *Nr4a1^–/–^* mice grafts after transplantation. (**D**) Flow cytometry quantification of CM (live CD45^+^Ly6G^–^NK1.1^–^CD11b^+^SiglecF^–^CD24^–^Ly6C^hi^) recruited into the allograft after i.v. injection of PBS liposomes (PBS-lip) or clodronate liposomes (Clo-lip) in donor mice or using WT or *Nr4a1^–/–^* as donor lungs (*n* = 5). (**E**) Normalized counts per minute (CPM) of *Ccl2* in sorted donor NCM isolated from allografts 2 and 24 hours after transplant. (**F**) Relative *Ccl2* mRNA levels of human and mouse NCM isolated before and after reperfusion (*n* = 3). (**G**) CCL2 levels in blood from mice in **D** (*n* = 5). Graphs show means ± SD. The graph in **B** was analyzed by 2-way ANOVA followed by Sidak’s post hoc test. *WT versus *Nr4a1^–/–^*; ^#^WT 2 hours versus WT 2.5 hours. The graph in **E** was analyzed by 1-way ANOVA followed by Tukey’s post hoc test. Graphs in **C**, **D**, **F**, and **G** were analyzed by unpaired Student’s *t* test. ^#^*P* < 0.05, **P < 0.01; ***P < 0.001; ****P < 0.0001. Scale bar: 50 μm.

Donor origin intravascular pulmonary NCM produce CXCL2 and recruit recipient neutrophils into the allograft (6). CM are recruited from the host spleen immediately after reperfusion and mediate neutrophil extravasation into the transplanted lung by permeabilization of the pulmonary endothelium through the production of IL-1β (9). Host splenectomy abrogates recruitment of the CM, neutrophil extravasation, and the development of PGD (9). We found that anti-CCR2 treatment of the recipients significantly attenuated the number of CM recruited to the transplanted lung (Figure 1A). Since anti-CCR2 antibody treatment could potentially lead to the depletion of the recipient CM, we used *Ccr2^–/–^* recipient mice and, similarly, found a decrease in the number of CM in the lung allograft (Figure 1B). However, when we analyzed the levels of CM in the spleen of *Ccr2^–/–^* recipients, we found monocytopenia (Figure 1C), which could potentially explain the reduction in the CM recruitment to the lung allograft in these recipients (Figure 1B). Therefore, we neutralized the chemokine ligand for CCR2 (CCL2) in the recipients by injecting anti-CCL2 antibodies (17) prior to transplantation and found a significant reduction in the CM influx into the transplanted lung (Figure 1D). Next, since we described that donor NCM initiate neutrophil recruitment (6), we hypothesized that they also played a role in the recruitment of recipient CM from the spleen. Indeed, when we depleted donor NCM, either genetically using *Nr4a1^–/–^* donor lungs (Figure 2, A–D, and Supplemental Videos 1 and 2; supplemental material available online with this article; https://doi.org/10.1172/jci.insight.147282DS1) or pharmacologically using i.v. clodronate liposomes (Clo-lip) treatment in the donors (Figure 2D, Supplemental Figure 1, and Supplemental Videos 3 and 4), the recruitment of CM to the reperfused lungs was attenuated based on both 2-photon imaging (Figure 2, A–C, and Supplemental Figure 1) and multichannel flow cytometry (Figure 2D). Of note, the extravasation of recruited CM was not impacted by the presence of donor NCM (Figure 2C). Accordingly, we explored whether donor NCM produced CCL2 to recruit recipient splenic CM. Surprisingly, secondary analysis of our previously published RNA sequencing (RNA-seq) data set (6) (Figure 2E), as well as quantitative PCR (qPCR) of donor human and murine NCM isolated immediately following reperfusion (Figure 2F), revealed that donor NCM were not the source of CCL2 secretion. Nevertheless, depletion of donor NCM led to a decrease in the blood CCL2 levels following murine lung transplantation (Figure 2G). Therefore, we hypothesized that donor NCM led to the secretion of CCL2 in the donor lung through a secondary mechanism.

### Depletion of donor AM suppresses recruitment of recipient CM to the transplanted lung and neutrophil extravasation.

**Figure 3:**
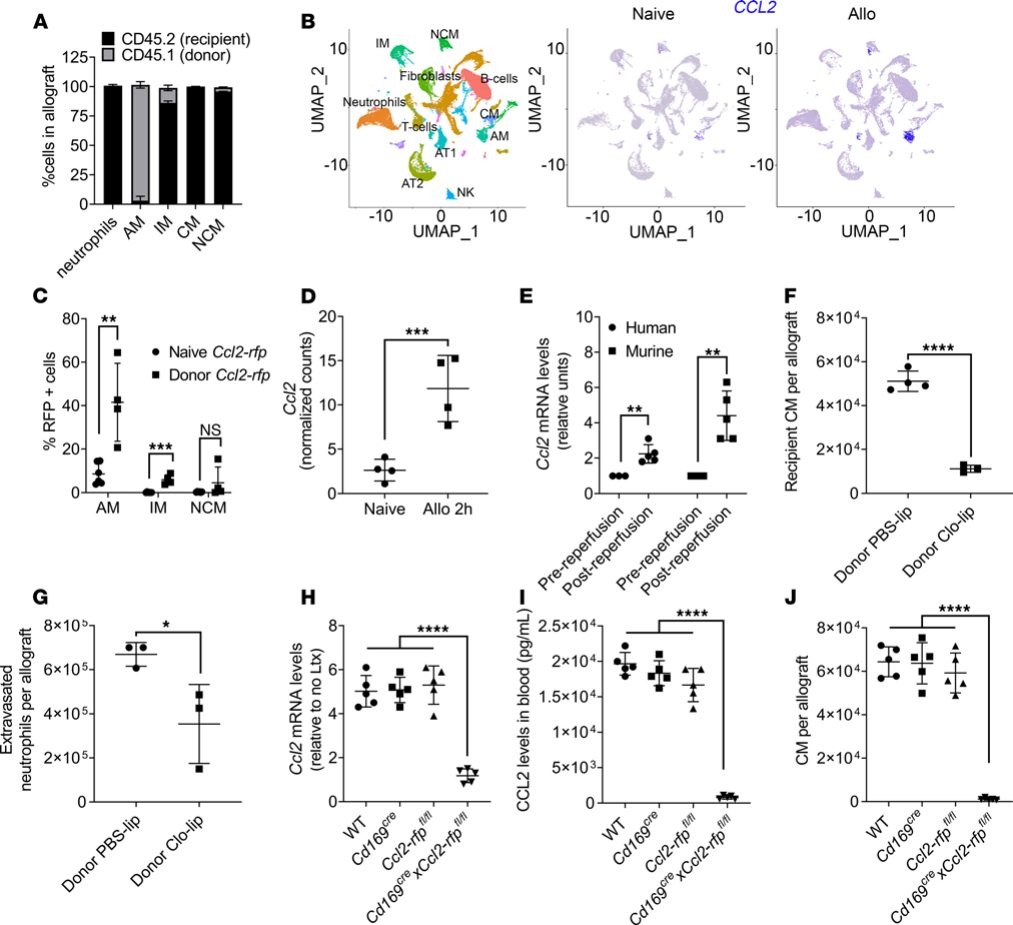
Depletion of donor alveolar macrophages (AM) suppresses recruitment of recipient classical monocytes (CM) to the transplanted lung. (**A**) Flow cytometry showing percentage of cells of donor (CD45.1) and recipient (CD45.2) origin in the allograft 24 hours after transplantation. Neutrophils were gated as live CD45^+^Ly6G^+^CD11b^+^CD24^+^SSC^hi^; AM were gated as live CD45^+^Ly6G^–^NK1.1^–^SiglecF^+^CD64^+^CD11c^+^; CM were gated as live CD45^+^Ly6G^–^NK1.1^–^CD11b^+^SiglecF^–^CD24^–^Ly6C^hi^; NCM were gated as live CD45^+^Ly6G^–^NK1.1^–^CD11b^+^SiglecF^–^CD24^–^Ly6C^lo^ (*n* = 3); and interstitial macrophages (IM) were gated as live CD45^+^Ly6G^–^NK1.1^–^CD11b^+^MHC II^+^CD11c^+^CD64^+^CD24^−^ (*n* = 3–6). (**B**) UMAP plot (left) and feature plots (middle and right) showing specific cell populations and expression of *Ccl2* in naive lungs and in allografts (Allo) 24 hours after transplant. (**C**) Flow cytometry showing percentage of RFP^+^ cells in lung allografts after transplantation of donor *Ccl2-rfp* grafts into WT recipient. AM and NCM were gated as in **A**. (**D**) Normalized counts per minute (CPM) of *Ccl2* in sorted mouse AM isolated from allografts 2 hours after transplant (*n* = 4). (**E**) Relative *Ccl2* mRNA levels of human and mouse AM isolated before and after reperfusion (*n* = 3–5). (**F**) Flow cytometry quantification of CM gated as in **A**, recruited into the allograft after intratracheal administration of PBS liposomes (PBS-lip) or clodronate liposomes (Clo-lip) in the donor mice (*n* = 4). (**G**) Flow cytometry quantification of extravasated neutrophils in the allograft gated as in **A**, after intratracheal administration of PBS-lip or Clo-lip in donor mice (*n* = 3). (**H**) Relative *Ccl2* mRNA levels in AM isolated from WT, *Cd169^Cre^*, *Ccl-rfp^fl/fl^*, and *Cd169^Cre^Ccl2-rfp^fl/fl^* allografts and compared with naive AM (*n* = 5). (**I**) Blood CCL2 levels after transplant combinations described in **H** (*n* = 5). (**J**) Flow cytometry quantification of CM gated as in **A**, after transplant combinations described in **H** (*n* = 5). Graphs show means ± SD. Graphs in **C**–**G** were analyzed by unpaired Student’s *t* test. Graphs in **H**–**J** were analyzed by 1-way ANOVA followed by Tukey’s post hoc test. *P < 0.05; **P < 0.01; ***P < 0.001; ****P < 0.0001.

Donor lungs are rapidly repopulated with recipient myeloid cells after transplantation, and 24 hours after transplant, only AM — and a few interstitial macrophages (IM) and NCM — remain of donor origin (Figure 3A and Supplemental Figure 2). Using the unbiased approach of transcriptional profiling with single cell RNA-seq (scRNA-seq), we compared lung myeloid cells from naive and donor lungs collected 24 hours after transplant. We found that *Ccl2* mRNA was mainly expressed by donor AM after lung transplantation (Figure 3B). We confirmed that donor AM are the dominant secretors of CCL2 following lung transplantation by using donor lungs from a reporter *Ccl2–*red fluorescent protein (*Ccl2-rfp*) mouse (18). Transplantation of donor *Ccl2-rfp* lungs into WT recipients allowed determination of CCL2 increase in the various donor cell lineages through the expression of RFP by flow cytometry. Following transplantation, we found that donor AM (and, to a much lesser extent, IM) are the predominant secretors of CCL2, as determined by an increase in the percentage of RFP-expressing cells (Figure 3C and Supplemental Figure 3). We confirmed the increase in *Ccl2* transcription in donor AM by secondary analysis of our previously published RNA-seq data set (19) (Figure 3D), as well as qPCR of donor human and murine AM isolated immediately following reperfusion (Figure 3E). To causally link CCL2 secretion by AM-to-CM recruitment, we depleted donor AM using Clo-lip (16) prior to transplantation and found a significant decrease in the recruitment of CM, as well as neutrophil extravasation, following transplantation (Figure 3, F and G). Since pharmacological depletion of donor AM can induce inflammation through cell death, we developed a conditional KO of *Ccl2* in the donor AM by crossing *Cd169^Cre^ and Ccl2-rfp^fl/fl^* mice (20). Donor lungs from *Cd169^Cre^Ccl2-rfp^fl/fl^* mice revealed lack of CCL2 production in the donor AM, reduction in CCL2 levels in pulmonary veins, and significantly decreased recruitment of recipient CM (Figure 3, H–J). Together, these data indicate that donor AM play a crucial role in the recruitment of recipient CM.

### CCL2 production by donor AM is dependent on IL-1β.

**Figure 4:**
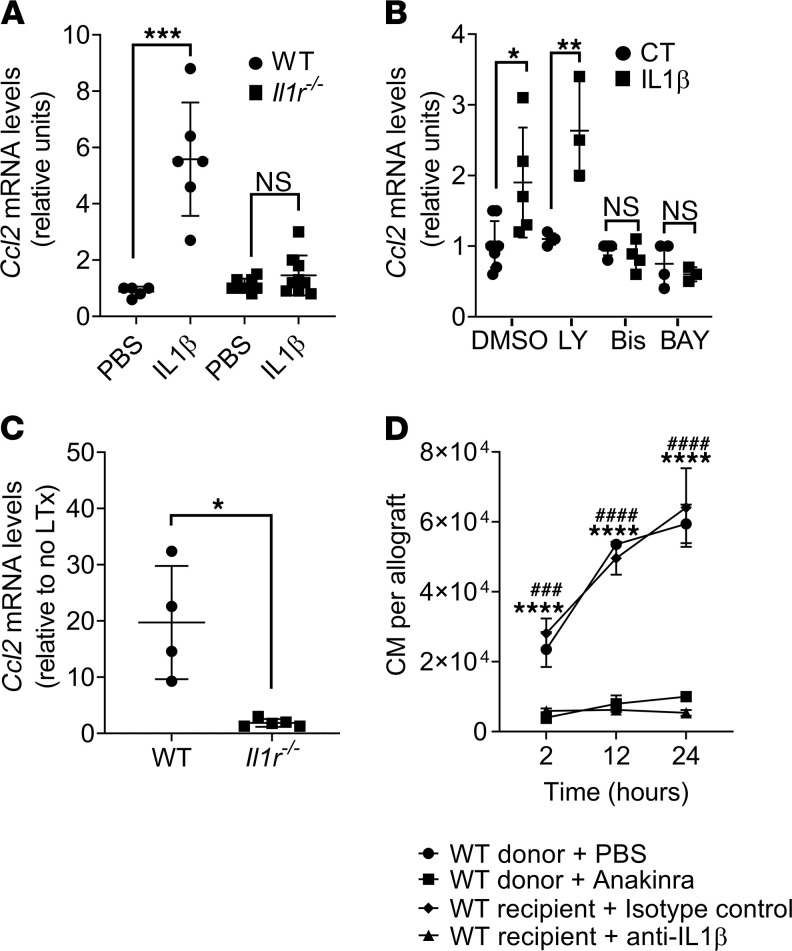
IL-1β is necessary for donor alveolar macrophages (AM) to produce CCL2. (**A**) AM were isolated from WT or *Il1r^–/–^* mice and incubated in vitro with PBS or 1 ng/μL of mouse recombinant IL-1β. *Ccl2* mRNA expression was measured by qPCR 24 hours after incubation (*n* = 6–8). (**B**) AM isolated from WT mice were incubated for 30 minutes in vitro with DMSO (control), 50 μM LY294002 (LY), 10 μM Bisindolylmaleimide-I (Bis), or 1 μM BAY11-7082 before adding 1 ng/μL of mouse recombinant IL-1β. *Ccl2* mRNA expression was measured by qPCR 24 hours after incubation (*n* = 3–7). (**C**) Donor AM were isolated from WT or *Il1r^–/–^* allografts 24 hours after transplant, and *Ccl2* mRNA expression was measured by qPCR (*n* = 4–5). (**D**) Flow cytometry quantification of CM (live CD45^+^Ly6G^–^NK1.1^–^CD11b^+^SiglecF^–^CD24^–^Ly6C^hi^) from allografts harvested at the indicated times from experiments in which donor lungs were treated with PBS or anakinra, or in which recipients were treated with isotype control and anti–IL-1β antibody (*n* = 3 per group). Graphs show means ± SD. Graphs in **A**–**C** were analyzed by unpaired Student’s *t* test. Graph in **D** was analyzed by 2-way ANOVA, followed by Sidak’s post hoc test. *PBS versus anakinra; ^#^isotype versus anti–IL-1β antibody. *P < 0.05; **P < 0.01; ***P < 0.001; ****P < 0.0001; ^###^*P* < 0.001; ^####^*P* < 0.0001.

Previous reports suggest that IL-1β can regulate the expression of CCL2 (21); therefore, we investigated whether AM expressed CCL2 in response to IL-1β both in vitro and in vivo. We found that AM freshly isolated from WT mice, but not *Il1r^–/–^* mice, expressed *Ccl2* in vitro when stimulated with IL-1β (Figure 4A). Binding of IL-1β to the IL-1R leads to cell signaling transduction events that culminate with NF-κB activation via PI3K- or PKC-dependent pathways (22–24). To determine whether CCL2 was dependent of PI3K- or PKC-dependent pathways, we treated freshly isolated AM with IL-1β in the presence or absence of specific inhibitors. Treatment with the PI3K inhibitor LY294002 did not alter IL-1β–induced *Ccl2* expression in AM, but Bisindolylmaleimide (PKC inhibitor) or BAY11-7082 (NF-κB inhibitor) both strongly suppressed *Ccl2* production (Figure 4B). Next, to determine the role of IL-1β in activating AM in vivo, we isolated AM from WT donor lungs transplanted into WT recipients and found increased *Ccl2* expression, which was significantly blunted in AM isolated from *Il1r^–/–^* donor mice transplanted into WT recipients (Figure 4C). We confirmed this observation by treating the donors with anakinra, an IL-1R inhibitor approved by the US Food and Drug administration (FDA) for human use. Donors treated with anakinra showed significantly decreased recruitment of CM at 2, 12, and 24 hours following transplantation (Figure 4D). Treatment of recipients with neutralizing antibodies against IL-1β revealed similar results at identical time points (Figure 4D).

### NLRP3 inflammasome dependent IL-1β release from donor NCM activates donor AM.

**Figure 5:**
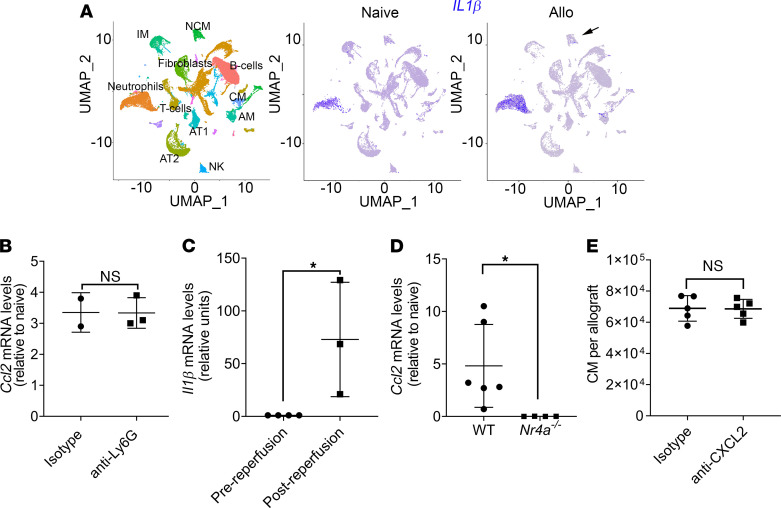
Nonclassical monocyte–derived (NCM-derived) IL-1β promotes CCL2 production by donor alveolar macrophages (AM). (**A**) UMAP plot (left) and feature plots (middle and right) showing specific cell populations and expression of *Il1β* in naive lungs and in allografts (Allo) 24 hours after transplantation. (**B**) *Ccl2* mRNA levels determined by qPCR in AM from the allograft after i.v. administration of IgG isotype or anti-Ly6G antibodies in recipient mice (*n* = 2–3). (**C**) Relative *Il1β* mRNA levels of mouse donor NCM isolated before and after reperfusion (*n* = 3–4). (**D**) *Ccl2* mRNA levels determined by qPCR in AM isolated WT or *Nr4a1^–/–^* lung allografts 24 hours after transplantation (*n* = 4–6). (**E**) Flow cytometry quantification of CM (live CD45^+^Ly6G^–^NK1.1^–^CD11b^+^SiglecF^–^CD24^–^Ly6C^hi^) recruited into the allograft after treatment of recipients with i.v. IgG isotype or anti-CXCL2 antibodies (*n* = 5). Graphs show means ± SD. Graphs were analyzed by unpaired Student’s *t* test.*P < 0.05.

The above data indicate that both donor NCM and donor AM are necessary for the recruitment of recipient CM, although only donor AM produced the chemokine CCL2 when stimulated by IL-1β. To determine whether donor NCM produce IL-1β, we analyzed the scRNA-seq from naive lungs and pulmonary grafts 24 hours after transplantation. We found that, in addition to NCM, neutrophils also express increased levels of *IL-1β* mRNA after lung transplantation (Figure 5A). To interrogate the role of neutrophils in IL-1β secretion, we depleted recipient neutrophils by administering anti-Ly6G antibodies (25) and analyzed *Ccl2* levels in donor AM following transplantation. We found that neutrophil depletion did not blunt the increased *Ccl2* expression in donor AM following reperfusion (Figure 5B). These results are indicative of a role for NCM in IL-1β production after lung transplant. We found increased *Il1β* mRNA in donor murine NCM isolated immediately following reperfusion by qPCR (Figure 5C). *Ccl2* expression in donor AM was abrogated after transplantation of *Nr41a^–/–^* donor lungs, indicating its dependence on IL-1β secretion by donor NCM (Figure 5D). Furthermore, recruitment of neutrophils and CM was an independent function of donor NCM, since administration of anti-CXCL2, which we have previously shown to abrogate neutrophil recruitment (6), did not change influx of recipient CM (Figure 5E).

**Figure 6:**
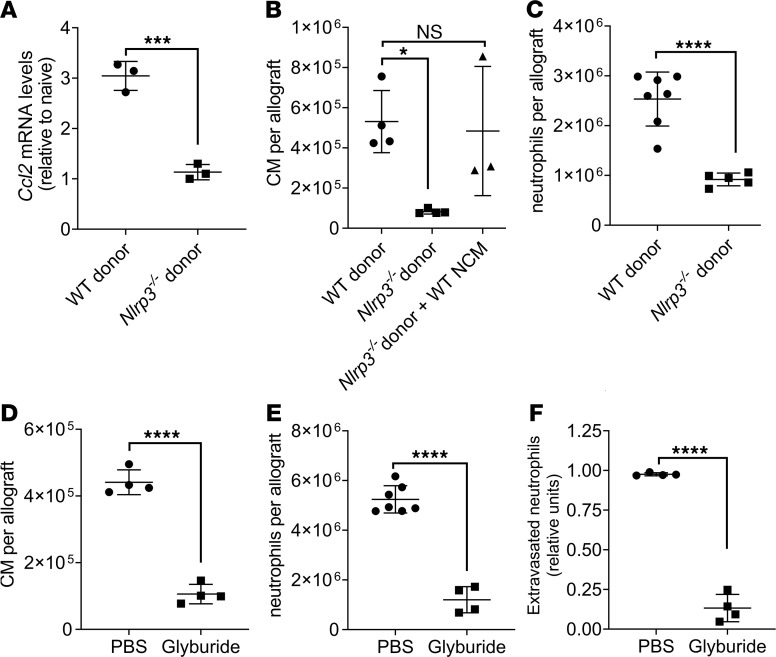
Nonclassical monocytes (NCM) produce IL-1β via NLRP3 inflammasome activation. (**A**) *Ccl2* mRNA levels determined by qPCR in AM isolated from WT or *Nlrp3^–/–^* lung allografts 24 hours after transplantation (*n* = 3). (**B**) Flow cytometry quantification of CM (live CD45^+^Ly6G^–^NK1.1^–^CD11b^+^SiglecF^–^CD24^–^Ly6C^hi^) recruited to allografts from WT, *Nlrp3^–/–^*, or *Nlrp3^–/–^* reconstituted with WT NCM donor mice (*n* = 3–4). (**C**) Flow cytometry quantification of neutrophils (live CD45^+^Ly6G^+^CD11b^+^CD24^+^SSC^hi^) recruited into WT of *Nlrp3^–/–^* allografts 24 hours after transplantation (*n* = 5–7). (**D**) Flow cytometry quantification of CM gated as in **C**, recruited into the allograft after donor treatment with 50 μg/g body weight glyburide (*n* = 4). (**E**) Flow cytometry quantification of neutrophils gated as in **B**, recruited into the allograft after i.p. administration glyburide in the donor mice (*n* = 4–7). (**F**) Flow cytometry quantification of extravasated neutrophils in the allograft gated as in **B**, after donor treatment with glyburide (*n* = 4). Graphs show means ± SD. Graph in **C** was analyzed by 1-way ANOVA, followed by Tukey’s post hoc test. All other graphs were analyzed by unpaired Student’s *t* test. *P < 0.05; ***P < 0.001; ****P < 0.0001.

The assembly of the NLRP3 inflammasome leads to caspase 1–dependent release of the IL-1β (26). Consistently, donor lungs from *Nlrp3^–/–^* mice showed reduced *Ccl2* expression by donor AM, and CM and neutrophil recruitment into the lung allograft (Figure 6, A–C). CM recruitment was restored after reconstitution of *Nlrp3^–/–^* donor lungs with WT NCM prior to transplantation (Figure 6B). To achieve clinical relevance, we treated murine donors with glyburide, a sulfonylurea that strongly inhibits the NLRP3 inflammasome (27). Treatment of donor lungs with glyburide resulted in significantly attenuated CM and neutrophil recruitment, as well as neutrophil extravasation following transplantation (Figure 6, D–F).

## Discussion

Inflammatory CCR2^+^ CM are recruited to the lung in response to injury, where they have diverse functions. Recruitment of these CM is a critical step in the development of lung transplantation ischemia-reperfusion injury (2, 9). CM facilitate extravasation of neutrophils into reperfused lungs and are central to the pathogenesis of PGD (3). In addition to PGD, they have been shown to play a crucial role in the development of alloimmunity (13). CCR2^+^ CM also have the ability to differentiate into profibrotic AM that may drive lung fibrosis (14). The inflammatory monocytes originate in the BM and migrate to the spleen, where they are stored for rapid deployment (10). While the egress of CM from the BM has been shown to be dependent on the expression of CCR2 (12, 28), the ligand CCL2 (also known as MCP-1) is necessary for their migration to the site of lung inflammation (29). These observations may be more generalizable, since CCR2^+^ monocytes promote the transendothelial migration of neutrophils in murine models of arthritis (30). Reports using *Ccr2*-deficient mice have also demonstrated that CCR2 signaling contributes to myocardial, renal, and cerebral ischemia-reperfusion injury (12, 18). Therefore, inhibiting the migration of CM into the transplanted lungs offers several benefits, such as amelioration of ischemia-reperfusion injury and posttransplant allograft dysfunction.

We have previously shown that donor origin NCM retained in the pulmonary vasculature play important roles in the recruitment of neutrophils. Depletion of donor NCM not only attenuated recruitment of recipient neutrophils, but also of recipient-derived CM (Figure 2). Because the recruitment of CM was dependent on CCL2, we hypothesized that, in addition to secreting the neutrophil chemoattractant CXCL2, donor NCM also released CCL2. Surprisingly, we found that donor NCM did not upregulate CCL2; therefore, we turned to an unbiased transcriptomic approach that revealed that donor AM were the predominant sources of *Ccl2*, an observation confirmed by the increase of RFP in AM from *Ccl2-rfp* donor lungs and direct *Ccl2* measurement in donor AM, as well as genetic and pharmacologic depletion of donor AM. While we also found some expression of CCL2 in donor IM, these cells are few, as they are almost completely replaced by cells of recipient origin by 24 hours after transplant. Furthermore, selective pharmacological depletion of AM or conditional deletion of *Ccl2* in the AM, using *Cd169^Cre^Ccl2-rfp^fl/fl^* donor mice, led to a significant reduction of *Ccl2* levels in the recipient, reduced CM recruitment, and protection from PGD.

IL-1β is a proinflammatory cytokine implicated in a wide range of physiological processes, including the initiation and orchestration of the inflammatory response to pathological inflammatory conditions (31). IL-1β is crucial for a variety of host responses to injury (32), including hepatic (33) and renal (34) ischemia-reperfusion injury. Elevated levels of IL-1β in donor lungs undergoing ex vivo lung perfusion have been proposed as a biomarker for PGD following transplantation (35–37) and were associated with increased alveolar neutrophils in a murine model of ischemia-reperfusion induced by hilar clamping (38). We found that AM produced CCL2 in response to IL-1β through a PKC- and NF-κB–dependent but PI3K-independent pathway. However, we recognize that in the lung, CCL2 can be produced by both stromal cells and hematopoietic cells (39, 40). However, our data suggest that stromal cells may not play an eminent role in CCL2 release in our lung transplant model.

The secretion of IL-1β from NCM initiated a signaling cascade that promoted the release of CCL2 from donor AM and the recruitment of CM. However, it is possible that cells of recipient origin may also contribute to IL-1β production. To this end, the transcriptional analysis revealed that recipient neutrophils also upregulated *Il1β*. However, depletion of neutrophils, at least at the initial time points following transplantation, did not alter the *Ccl2* expression in donor AM. We, nevertheless, recognize that it is possible that neutrophils can perpetuate the activation of donor AM after they are recruited to the allograft by donor NCM. In the clinical context, differentiating the relative contribution of donor NCM and recipient neutrophils in IL-1β production may not be relevant since neutrophil recruitment is dependent on donor NCM and can be abrogated by targeting the donor NCM prior to transplantation. Our prior studies have also shown that CCR2^+^ monocytes can produce large amounts of IL-1β after being recruited to the lung grafts (9). We postulate that the IL-1β derived from the recruited CCR2^+^ CM can also create a self-propagating circuit to recruit more CM through ongoing activation of donor AM. Since these recruited CM also contribute to the development of alloimmunity (13), our studies may provide an immunological link between severe forms of PGD and chronic allograft rejection (1, 20). Our unbiased transcriptomic analysis combined with genetic loss- and gain-of-function experiments in a clinically relevant murine model of lung transplantation show causality between IL-1β secretion by donor NCM and activation of AM, while indicating that any contribution of IL-1β by other myeloid cells is downstream of donor NCM. Collectively, our previously published work (6, 9) and the current study demonstrate that donor NCM serve a dual role in recruiting host neutrophils, as well as CM. The recruitment of host neutrophils by donor NCM occurs directly through MyD88/TRIF-dependent secretion of CXCL2 (6). However, the recruitment of host CM occurs indirectly by donor NCM-dependent production of CCL2 by donor AM. Recruitment of neutrophil and CM are independent of each other, since neutrophil depletion did not alter CCL2 levels and neutralization of CXCL2 did not reduce CM recruitment.

IL-1β is produced as an inactive 31 kDA precursor, pro–IL-1β, and processing and secretion of biologically active IL-1β is inflammasome dependent (41, 42). The best characterized inflammasome is formed by the cytosolic NLRP3 (43). Using single cell transcriptomic profiling, we observed that *Il1β* is upregulated in donor NCM following transplantation. Causal studies in this report show that the NLRP3 inflammasome is responsible for the NCM-dependent activation of the donor AM, leading to the secretion of CCL2. Although our results using the reconstitution of genetically deficient mice are compelling, it is also possible that neutrophil-derived serine proteases — including proteinase 3 (PR3), elastase, and cathepsin-G — can cleave secreted pro–IL-1β (32, 44, 45), or that other known inflammasomes such as NLRP1, NLRC4, and absent in melanoma 2 (AIM2) (46) lead to the production of IL-1β from the NCM (47–55). Models described in our report could be used for future studies to determine the role of these alternate mechanisms, as well as activation of downstream NLRP3 inflammasome proteins in NCM by using NCM from *A*sc^−/−^ and Caspase-1^−/−^ mice (48, 50–54).

The findings in this study are particularly important, since they identify common pathways to attenuate the recruitment of damaging neutrophils as well as the recruitment of CM. Depletion of donor NCM by agents such as bisphosphonates has the potential for not only preventing neutrophil recruitment, but also CM. Clodronate, a first-generation bisphonate, is approved for human use in Europe and Canada. Alternatively, IL-1β–IL-1R interactions could be targeted therapeutically, as we described with the use of anakinra (IL-1R inhibitor) and neutralizing antibodies against IL-1β, to serve the dual purpose of not only preserving endothelial integrity (9), but also attenuating the recruitment of inflammatory splenic monocytes. NLRP3 inflammasome inhibition is another alternative strategy to abrogate the interaction between the NCM and AM. Our experiments using the inflammasome inhibitor glyburide are attractive, as it should be feasible to treat the donor lung prior to transplant to avoid toxicity in the recipient. Since glyburide is FDA approved, positive findings could be potentially translated to human lung transplantation.

In this report, wherever possible, we complemented our genetic studies in mice with preclinical studies using pharmacologic agents. We perform detailed analyses of small biopsies of tissues from the donor lung obtained in the operating room before and after reperfusion to confirm the relevance of our findings for our patients. We have used both allogeneic and syngeneic models in our study. However, in the majority of the experiments, we have used allogeneic murine transplants, since they are more relevant to the clinical transplantation. Furthermore, while we acknowledge that allogeneic transplants induce adaptive immunity, the goal of the present study was not to study the adaptive immune response. We have previously shown that allogeneic and syngeneic murine lung transplants are comparable when studying ischemia-reperfusion injury/PGD or neutrophil and monocyte trafficking (6, 9). Our studies also show a paracrine signaling between donor cells, resident intravascular monocytes and tissue-resident extravascular macrophages, to recruit inflammatory monocytes from the spleen. This complex interplay between the donor and recipient cells is exciting, since drugs inhibiting these pathways are already approved for use in humans. Furthermore, since therapies targeting signaling in these donor cells could be potentially administered to donor lungs prior to transplantation, toxicity to the recipient can be minimized. In summary, we provide evidence that IL-1β–dependent activation of donor AM by donor NCM leads to recruitment of host CM, which are responsible for neutrophil extravasation and pathogenesis of PGD. Since inflammasome inhibition, depletion of donor NCM, IL-1β, or IL-1R antagonism abrogate recruitment of CM, as well as PGD, and are feasible using FDA-approved compounds, our findings may have potential for clinical translation.

## Methods

### Human samples

Lung graft samples were obtained both before and after reperfusion. Prereperfusion samples were obtained at the conclusion of cold ischemic storage (defined as the period between cooling of organs inside the donor to extraction of the organs from the cold storage transport medium for implantation into the recipient). Postreperfusion samples were obtained after 15 minutes of reperfusion. Donor NCM were identified from fresh specimens using multicolor flow cytometry, using our previously described protocols (16), and were isolated 15 minutes following reperfusion. Bronchoscopic bronchoalveolar lavage fluid (BALF) was used to collect donor AM. Briefly, BALF was obtained in the times above indicated, centrifuged (10 minutes, 400*g*, 4°C), and RBC lysed, and AM were collected. A total of 7 lung grafts were studied.

### Mice and procedures

#### Mice.

Male WT C57BL/6J (B6), BALB/c, BALB/c 45.1, *Ccr2^–/–^*, *Ccr2-gfp*, *Nr4a1^–/–^*, *Ccl2-rfp^fl/fl^*, *Cd169^Cre^*, *Il1r^–/–^,* and *Nlrp3^–/–^* mice were obtained from The Jackson Laboratory. Anakirna (100 μg/g body weight, Swedish Orphan Biovitrum) and anti–IL-1β (50 μg/g body weight, clone AF-401, R&D Systems) were used for IL-1β–IL-1R antagonism. Donors were treated with glyburide (50 μg/g body weight, i.p., MilliporeSigma) for 3 days before lung harvest. We used the dose of glyburide of 50 μg/g body weight according to the published literature; prior studies have used a broad range of glyburide from 2.5 μg/g (56) to 500 μg/g (27). We opted for a dose that was in the mid-range, as shown in multiple prior reports (57, 58). All mice were maintained in a specific pathogen–free facility at the Center for Comparative Medicine at Northwestern University and used for the described experiments between the ages of 9–14 weeks and between 24–28 grams of body weight. A total of 400 mice were used in these studies.

#### Mouse lung transplantation.

Orthotopic murine left lung transplantation was performed as previously described (6). All lung transplants performed in this study were allogeneic (except for Figure 2A, which were syngeneic using B6 mice), using B6 and BALB/c background mice. Unless specified otherwise, lungs were harvest 24 hours after transplantation. Briefly, donor mice were anesthetized with a mixture of xylazine (10 mg/kg, Akorn Pharmaceutical) and ketamine (100 mg/kg, Covetrus). Donor lungs were flushed through the pulmonary artery with 3 mL of saline solution, and the heart-lung block was excised and kept in cooled (4°C) preservative solution. The bronchus, pulmonary vein, and artery were dissected and prepared for anastomosis. A customized cuff made of a Teflon i.v. catheter was applied to the vascular structures and secured with a 10-0 nylon ligature. After placement of a microvessel clip on the bronchus to avoid airway infiltration with preservative solution, the graft was stored at 4°C for a period of 90–120 minutes prior to implantation. Recipient mice received s.c. buprenorphine (0.1 mg/kg) 30 minutes prior to the thoracic surgical incision and every 6 hours as needed after the transplant procedure. Recipient mice were intubated, and a left-sided thoracotomy was performed on each mouse within the third intercostal space. The recipient’s native lung was gently clamped and pulled out of the thoracic cavity. The space between the artery, the vein, and the bronchus was dissected separately. The artery and vein were temporarily occluded using 8-0 nylon ligatures. The anastomoses were completed by securing each cuff with 10-0 nylon ligatures. The 8-0 ligatures were released (first vein and then artery), and the lung was inflated. The chest incision was closed, and recipients were separated from the ventilator when spontaneous respiration resumed. No antibiotics or immunosuppressive agents were used postoperatively in any groups.

#### CCL2, CXCL2, and cell depletion.

Clodronate-loaded liposomes and control phosphate-buffered saline liposomes were purchased from Clodronateliposomes.com. At specific time points, mice were injected intratracheally (50 L) or i.v. (200 L) with clodronate-loaded liposomes or control PBS-loaded liposomes to deplete AM or monocytes, respectively (59). Cytotoxic anti-CCR2 antibody (a gift from Steffen Jung, The Weizmann Institute of Science, Israel) was used for selective depletion of CCR2^+^ CM as previously described (50 μg i.v. 24 hours prior to lung transplant) (6). CCL2 antibody (50 μg/mouse, clone AF-479, R&D Systems) was injected i.v. to neutralize CCL2 (17). CXCL2 antibody (50 μg/mice, clone AF-452, R&D) was injected i.v. to neutralize CXCL2. Neutrophils were depleted by using Ly6G antibody (12.5 mg/Kg body weight, clone 1A8, BioLegend). Control mice were treated with the same amounts of IgG isotype control antibodies (R&D).

#### NCM reconstitution.

NCM were isolated as described before (6). Briefly, mouse lung tissue was digested, single cell suspensions were prepared, and NCM were sorted from WT mice using a FACSARIA 4-Laser Sorter. For reconstitution of *Nlrp3^–/–^* mice, 50,000–100,000 NCM were injected into *Nlrp3^–/–^* mice into the donor pulmonary artery of the donor murine lungs ex vivo, immediately prior to implantation of the lung.

### Flow cytometry and sorting

Human and mouse lung and mouse spleen tissue were digested, and single cell suspensions were prepared as previously described (6, 9). Cell suspensions underwent RBC lysis using Pharm Lyse buffer (BD Biosciences). Live/dead staining was performed in protein-free solution (HBSS) using fixable viability dye eFluor 506 (eBioscience), followed by incubation with FcR-blocking reagent (Miltenyi Biotec). Cells were then stained with the different antibodies (Table 1 and Table 2). NCM were sorted using a FACSARIA 4-Laser Sorter, and flow analysis of fixed samples was performed on a BD FACS Symphony A5-Laser Analyzer at the Northwestern University Robert H. Lurie Comprehensive Cancer Center Flow Cytometry Core facility. Acquired data were analyzed with FlowJo v10.6 (FlowJo).

### Two-photon microscopy

A custom built 2p microscope running ImageWarp acquisition software (A&B Software) was used for time-lapse imaging. Mice were anesthetized with an i.p. injection of ketamine (50 mg/kg) and xylazine (10 mg/kg) and maintained with administration of half doses every hour through the imaging process. Mice were intubated orotracheally and ventilated with room air at a rate of 120 breaths/minute and with a tidal volume of 0.5 mL. Left thoracotomy was performed to expose the left lung, and the lung was imaged using a custom-built chamber maintained at 37°C. Vetbond ring was used to attach the lung tissue to the bottom of the cover glass without direct pressure on the exposed lung. For time-lapse imaging of migration of CCR2^+^, we averaged 15 video-rate frames (0.5 seconds per slice) during the acquisition to match the ventilator rate and minimize movement artifacts. Each plane represents an image of 220 × 240 μm in the *x* and *y* dimensions. To visualize blood vessels, 20 μL of 655 nm nontargeted Qdots in 100 μL of PBS was injected i.v. prior to imaging (60–62). Two-photon microscopy images for Clo-lip experiments shown in Supplemental Figure 1 were acquired using a water immersion lens (Apo LWD 25× 1.10W DIC N2) on a Nikon A1R-MP+ multiphoton microscope system with a Coherent Chameleon Vision titanium sapphire laser. For each mouse, images of 512 × 512 μm in the *x* and *y* dimensions were acquired using an excitation wavelength tuned at 920 nm. To visualize blood vessels, 100 μL (5 mg/mL) of dextran tetramethylrhodamine in PBS were injected i.v. 5 minutes prior to imaging. Images were processed and analyzed using NIS-Elements NIS.ai (Nikon), Imaris (Bitplane), and NIH ImageJ software.

### Secondary transcriptomic analysis

#### RNA-seq.

RNA isolation and sequencing procedures are detailed in our previous manuscript (6). For all analyses, normalized tables of counts were generated from raw counts using the EdgR package (version 3.18.1). Filtering for low counts was performed by excluding genes, which have the expression from more than half of all the samples.

### scRNA-seq

Single cell suspensions from naive lungs and lung grafts 24 hours after transplantation were prepared as described above for flow cytometry with slight modifications. Grafts were removed and digested with 3 mL dispase (Corning) with DNase I (MilliporeSigma) and were gently teased using forceps into small (1–2 mm) fragments, followed by incubation at room temperature with gentle agitation for 30 minutes. The resulting suspension (in DMEM + 5% FBS) was passed through 70 μm cell strainer; erythrocytes were lysed and filtered through 40 μm cell strainers. Cells were counted using acridine orange/propidium (AO/PI) and Cellometer K2 (Nexcelom), and cell viability exceeded 90%. Single cell 3’ RNA-Seq libraries were prepared using Chromium Single Cell V2 Reagent Kit and Controller (10x Genomics). Libraries were assessed for quality (TapeStation 4200, Agilent Technologies) and then sequenced on NextSeq 500 or HiSeq 4000 instruments (Illumina). Initial data processing was performed using the Cell Ranger version 2.0 pipeline (10x Genomics), and reads were mapped to mm10 version of the mouse genome, Ensemble build 84. Downstream scRNA-seq analysis was performed using Seurat Package version 3.1.0 following the standard workflow posted on the Satija lab website (https://satijalab.org/seurat/) (63). Specifically, SCTransform with an anchor-based integration approach was used to integrate 4 different samples from 2 conditions. Cell types were identified using both manual annotations based on positive markers from FindAllMarkers function in Seruat pipeline and a supervised annotation tool, SingleR (64). The RNA-seq data can be found in the GEO repository (geo166679).

### mRNA isolation and qPCR

Total RNA was extracted using miRNeasy Mini kit (Qiagen). cDNA was synthesized from 5–250 ng of total RNA by using a qScript cDNA Synthesis kit (Quanta Biosciences), and mRNA expression was determined by qPCR using iTaq Universal Probes Supermix (Bio-Rad). Relative expression of the transcripts was determined according to the ΔΔCt method using actin or rpl19 as reference for normalization. The following primers were used for mouse samples*: Ccl2-F,* 5′ - GCATCCACGTGTTGGCTCA - 3′*; Ccl2-R*, 5′ - CTCCAGCCTACTCATTGGGATCA - 3′; *β-actin–F*, 5′ - CACCACACCTTCTACAATGA - 3′*;* *β-actin–R*, 5′ - GTCTCAAACATGATCTGGGT - 3′*; Rpl19-F*, 5′ - GAAATCGCCAATGCCAACTC - 3′; *Rpl19-R*, 5′ - CTTCAGGTACAGGCTGTGATAC - 3′; *Il1β-F*, 5′ - GGCAGGCAGTATCACTCATT - 3′; and *Il1β-R*, 5′ - CCCAAGGCCACAGGTATTT - 3′. The following primers were used for human samples: *Ccl2-F*, 5′ - GCAGAAGTGGGTTCAGGATT - 3′; *Ccl2-R*, 5′ - GGGTAGAACTGTGGTTCAAGAG - 3′; *β-actin–F*, 5′ - GAAGTCCCTTGCCATCCTAAA; and *β-actin–R*, 5′ - GTCTCAAGTCAGTGTACAGGTAAG - 3′.

### ELISA

Human and mouse CCL2 ELISA were performed using commercially available kits accordingly to the manufacturer’s instructions (Thermo Fisher Scientific; Human MCP1 ELISA kit [BMS281] and MCP-1 Mouse ELISA Kit [BMS6005]).

### Compartmental lung i.v. staining for neutrophils extravasation

I.v. and intratracheal procedures were performed utilizing an adaptation of previously described methods (16, 19). Briefly, 6 μg of APC-conjugated anti-CD45 in 100 μL of sterile PBS was injected i.v. and allowed to circulate for 3 minutes prior to euthanasia with an overdose of Euthasol. Tracheostomy was performed. The vena cava was transected, and the right ventricle was flushed with 10 mL of HBSS to wash unbound antibody.

### AM isolation and in vitro experiments

AM were isolated as previously described (16). Briefly, BALF from mice was obtained by performing a tracheostomy and lavaging with 2 mM EDTA-PBS solution (1 mL, 10 times per mouse), followed by centrifugation (10 minutes, 400*g*, 4°C) and RBC lysis. AM were resuspended in RPMI media supplemented with 10% FBS and antibiotics. Between 50,000 and 100,000 cells were plated in 24-well plates, allowed to rest for 2 hours, and incubated with 1 ng/μL mouse recombinant IL-1β (Thermo Fisher Scientific) in the absence or presence of various antagonists (30 minutes before adding IL-1β): 50 μM LY294002 (Thermo Fisher Scientific), 10 μM Bisindolylmaleimide I (Calbiochem), and 1 μM BAY11-7082 (Calbiochem). After 24 hours, media was discarded, AM was resuspended in RLT (Qiagen), and RNA was extracted as described above.

### Statistics

Data analysis was performed using Prism 8 (GraphPad Software Inc.). Results are expressed as mean ± SD, and the *n* values for each data set are provided in the figure legends. Statistical significance was assessed by 2-tailed Student’s *t* test, 1-way ANOVA followed by Tukey’s post hoc test, or 2-way ANOVA followed by Sidak’s post hoc test. A *P* value less than 0.05 was considered significant.

### Study approval

All procedures were approved by the IACUC (IS00002248) at Northwestern University. Animals received humane care in compliance with the *Guide for the Care and Use of Laboratory Animals* (National Academies Press, 2011) and the Principles of Laboratory Animal Care formulated by the National Society for Medical Research. Human protocols were approved by the IRB at Northwestern University Feinberg School of Medicine (STU00106589). All study subjects provided written informed consent prior to participation in the study.

## Author contributions

CK and EL contributed to conceptualization, study design, methodology, data collection, validation, formal analysis, and manuscript writing. Authorship was assigned by alphabetical order. WY, FLNS, and ZR contributed to formal analysis and methodology. QW, MA, WL, SR, MLA, HS, and MEK conducted experiments. XL, GRSB, MQ, EC, HAV, AS, TM, and DK contributed to formal analysis, visualization, and manuscript writing. AB contributed to conceptualization, methodology, validation, formal analysis, investigation, resources, data curation, writing, visualization, supervision, project administration, and funding acquisition.

## Supplementary Material

Supplemental data

Supplemental Video 1

Supplemental Video 2

Supplemental Video 3

Supplemental Video 4

## Figures and Tables

**Table 1:**
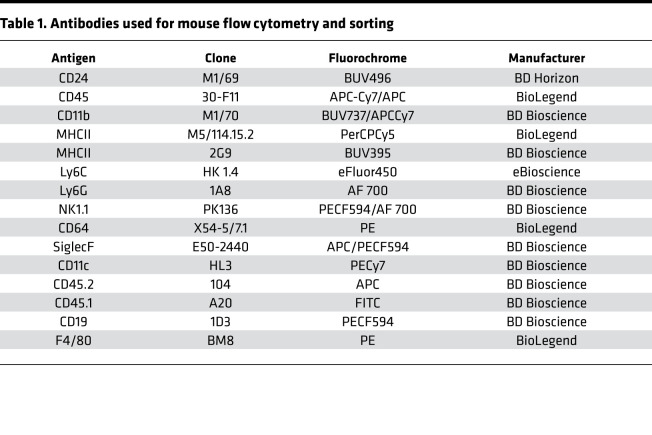
Antibodies used for mouse flow cytometry and sorting

**Table 2:**
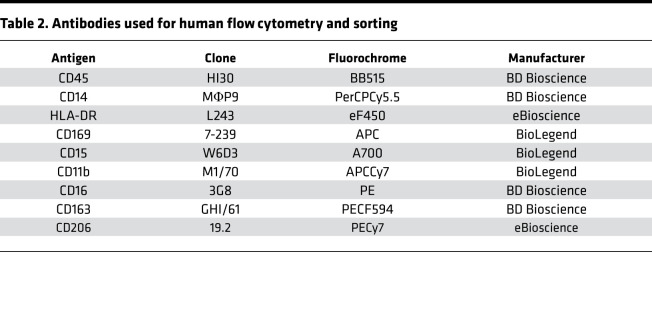
Antibodies used for human flow cytometry and sorting

## Version 1 02/23/2021

In-Press Preview

## Version 2 03/22/2021

Electronic publication
