# Supplementary material for: Crosstalk between nonclassical monocytes and alveolar macrophages mediates transplant ischemia-reperfusion injury through classical monocyte recruitment
Source: JCI Insight. 2021 Mar 22;6(6):e147282. doi: 10.1172/jci.insight.147282 (PMC8026186; doi:10.1172/jci.insight.147282)
Supplement: Supplemental data [file jciinsight-6-147282-s178.pdf]

Supplemental Figure 1. Allografts from donor administered with Clodronate liposomes have decreased recruitment of classical monocytes. Intravital two-photon imaging at 2 hours through 2 hours and 30 minutes after reperfusion. (A) Representative still images of donor lungs from PBS-liposomes (PBS-lip) and Clodronate-liposomes (Clo-lip) treated allografts. Green, CCR2-GFP (recipient); red, dextran blood vessels. (B) CCR2-GFP cell density calculated using NIH ImageJ software in PBS-lip and Clo-lip injected mice. Data was analyzed by Unpaired Student's t-test. n.s: not significant; $^{**}p<0.01$.

Supplemental Figure 2. Cells of donor and recipient origin in allograft 24 hours post-transplant. Allogeneic lung transplant was performed using wild-type C57BL/6J CD45.2 as recipient and BALB/c CD45.1 as donor. Flow cytometry was performed in naïve lungs as well as in the allograft. Neutrophils were gated as live $CD45^{+}Ly6G^{+}CD11b^{+}CD24^{+}SSC^{hi}$; Alveolar macrophages (AM) were gated as live $CD45^{+}Ly6G^{-}NK1.1^{-}SiglecF^{+}CD64^{+}CD11c^{+}$; Interstitial macrophages (IM) were gated as live $CD45^{+}Ly6G^{-}NK1.1^{-}CD11b^{+}MHCII^{+}CD11c^{+}CD64^{+}$ $CD24^{-}$; Classical monocytes (CM) were gated as live $CD45^{+}Ly6G^{-}NK1.1^{-}CD11b^{+}Siglec^{-}CD24^{-}Ly6C^{+}$; Non-classical monocytes (NCM) were gated as live $CD45^{+}Ly6G^{-}NK1.1^{-}CD11b^{+}Siglec^{-}CD24^{-}$ $Ly6C^{+}$.

Supplemental Figure 3. RFP positive cells of donor origin in allograft 24 hours post-transplant. Allogeneic lung transplant was performed using wild-type *Ccl2-rfp* as wild-type. Flow cytometry was performed to determine RFP positive cells. Figure shows representative gating of alveolar macrophages (AM; live $CD45^{+}Ly6G^{-}NK1.1^{-}SiglecF^{+}CD64^{+}CD11c^{+}$), interstitial macrophages (IM; live $CD45^{+}Ly6G^{-}NK1.1^{-}CD11b^{+}MHCII^{+}CD11c^{+}CD64^{+}$ $CD24^{-}$) and non-classical monocytes (NCM; live $CD45^{+}Ly6G^{-}NK1.1^{-}CD11b^{+}Siglec^{-}CD24^{-}Ly6C^{+}$) separated by RFP expression.

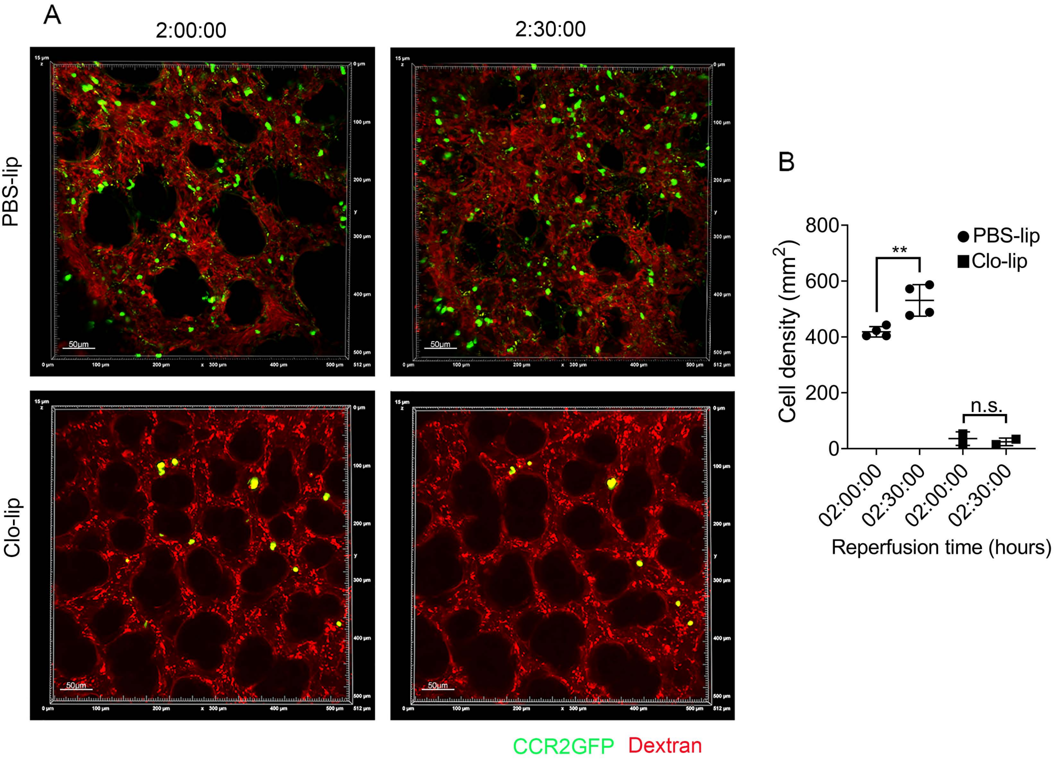

Supplemental Figure 1

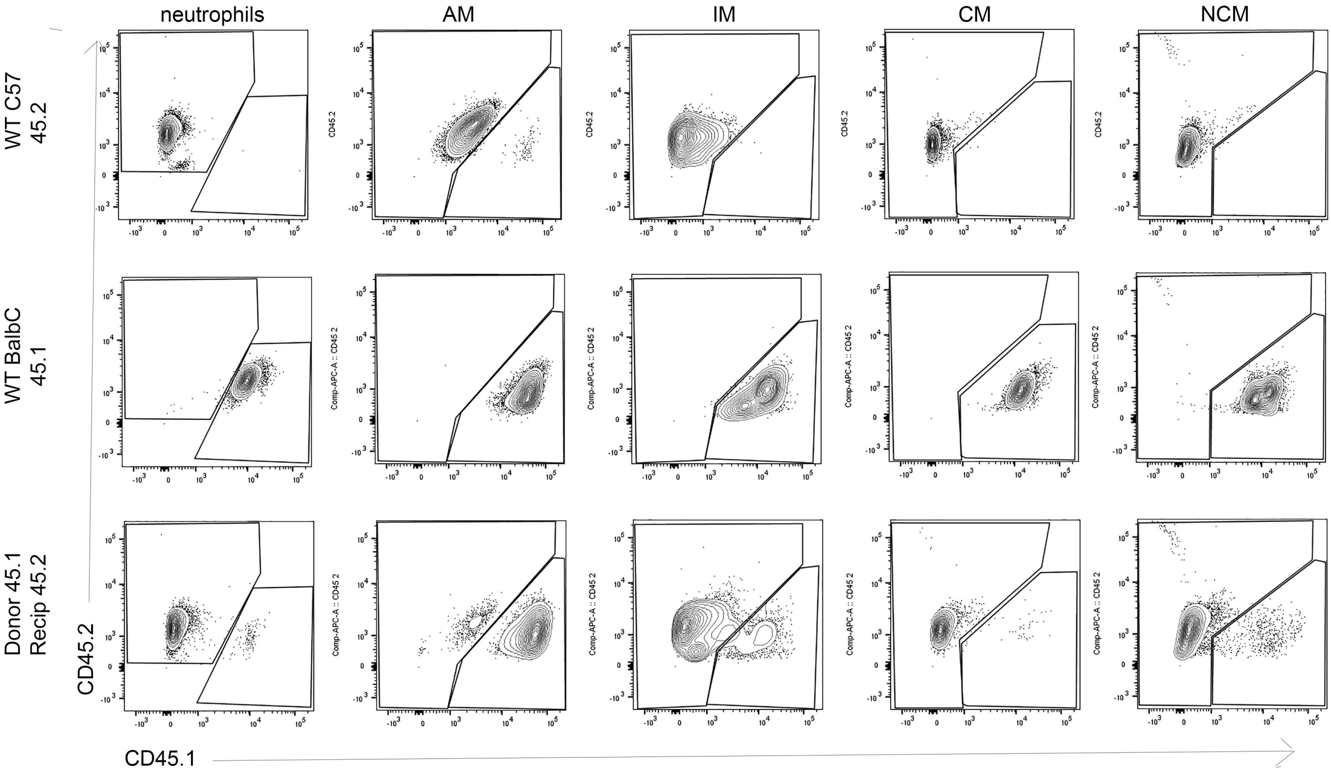

Supplemental Figure 2

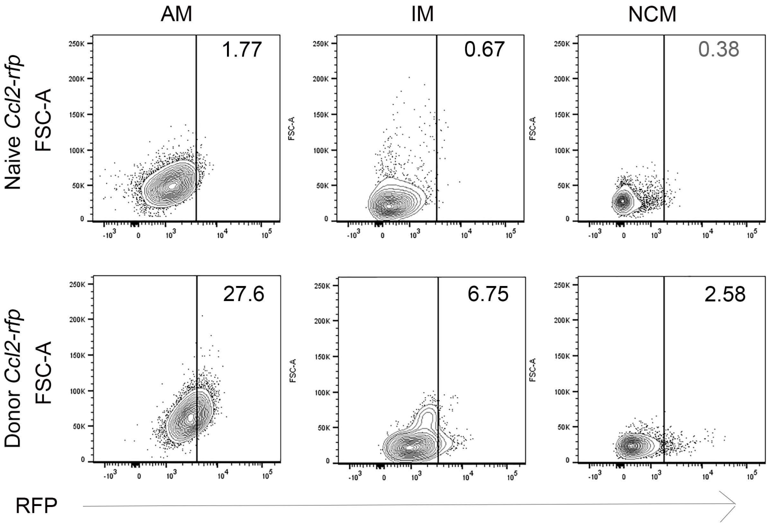

Supplemental Figure 3
